# Comparative Evaluation of Dentinal Defects After Root Canal Preparation Using Various Nickel Titanium Files: An In Vitro Study

**DOI:** 10.7759/cureus.38829

**Published:** 2023-05-10

**Authors:** Renu Batra, Ankita Dixit, Anushree Tiwari, Amit Kumar, Shagun Sinha, Sheetal Badnaware, Ramanpal Singh

**Affiliations:** 1 Department of Conservative Dentistry and Endodontics, K.M. Shah Dental College and Hospital, Sumandeep Vidyapeeth Deemed to be University, Vadodara, IND; 2 Department of Pedodontics and Preventive Dentistry, Ahmedabad Dental College and Hospital, Ahmedabad, IND; 3 Clinical Quality and Value, American Academy of Orthopaedic Surgeons, Rosemont, USA; 4 Department of Dentistry, All India Institute of Medical Sciences, Patna, IND; 5 Department of Pedodontics and Preventive Dentistry, Institute of Dental Studies and Technologies, Modinagar, IND; 6 Department of Pedodontics and Preventive Dentistry, Faculty of Dental Sciences, Institute of Medical Sciences (IMS), Varanasi, IND; 7 Oral Medicine and Radiology, New Horizon Dental College and Research Institute, Chhattisgarh, IND

**Keywords:** nickel titanium files, dentinal defect, waveone gold, protaper next, hyflex edm files

## Abstract

Aim: This study set out to compare the damage done to dentin by three distinct titanium file brands - the Hyflex EDM, the ProTaper Next, and the Waveone Gold Nickel - in order to draw conclusions about which one is the most effective.

Materials and methods: Forty-first premolars in the mandible with straight canals and single roots were instrumented using Hyflex EDM, Waveone Gold, and Protaper Next. Dentinal flaws after endodontic treatment were studied by sectioning specimens using a hard tissue microtome and analyzing them under a stereomicroscope.

Results: There was no discernible variation between the groups in the coronal third (p=0.312) or apical third (p=0.076). Hyflex EDM and Protaper Next differed significantly in the middle portion of the tape (p=0.016). The Hyflex EDM sample had the fewest cracks. There was no statistically significant difference between Hyflex EDM and Waveone Gold; however, Hyflex EDM had fewer fractures in the middle third of the sample than Waveone Gold did.

Conclusion:Electric discharge machining (EDM) files made from Hyflex proved to be far superior to their Protaper Next and Waveone Gold counterparts as they induced the fewest cracks in the middle third of the root dentin.

## Introduction

Root canal preparation using chemo-mechanical techniques aims to clean the whole canal system while still preserving the canal's natural shape. New rotary nickel-titanium instruments allow for more precise cleaning and contouring with fewer abrasions to the dentin. Root canal-treated teeth are at risk for problems such as vertical root fractures (VRF) and microcracks in the dentin [1]. VRFs are a frequent consequence of mechanical root canal preparation and may result in tooth loss. Dentinal flaws, such as micro-cracks that might cause vertical root fractures, are generally agreed upon as something to be avoided, yet there is currently inconclusive data to support this position.

The use of rotary and reciprocating devices has been linked to the development of microcracks in dentin [2,3], according to a number of microscopic investigations. Chemo-mechanics root canal preparation makes an effort, in part, to prevent the microcracking of the dentin that could happen from the use of rotating and reciprocating files. The term EDM refers to electrical discharge machining. This is a distinctive method of processing files that utilizes electric discharge machining. In the EDM manufacturing process, workpieces are machined by creating a potential between the workpiece and the tool. The material's surface melts and evaporates as a result of the sparks produced during this process. This gives the new Niti files their distinctive surface and strengthens and increases the fracture resistance of the Hyflex EDM files [4]. The number of files used for cleaning and shaping during root canal treatment can be decreased without compromising the preservation of the root canal morphology thanks to this incredibly rare combination of flexibility and fracture resistance [4]. Glide path files created with HyFlex EDM can take on one of three different horizontal cross-sections: quadratic at the end, trapezoidal in the middle, or triangular at the beginning or end of the file. A single file with a 0.10 mm tip diameter and a 5% taper is used to create the HyFlex EDM glide path.

Except for the top 3 mm of the X1 file, which is square, the cross-section of all Protaper Next instruments has a distinctively off-centered rectangular shape. There are a variety of taper lengths and tip sizes available, including 17/.04, 25/.06, 30/.07, 40/.06, and 50/.06. (In the apicocoronal direction, X1 and X2 increase and then decrease; X3, X4, and X5 remain constant and then decrease.) Taper lock, screw-in, and torque may all be reduced with these design choices [5]. They have also been researched to improve flexibility and debris removal. Gold Wave One files are the most recent iteration of the Wave One format (Dentsply Maillefer). Changes were made to the size, shape, and cross-section of the files without disrupting their reciprocating motion. Alternately, a parallelogram with an offset and two cutting edges [6] was used as the cross-section for the file.

The ideal phase transition between martensite and austenite for creating a clinically superior metal to NiTi has been determined by engineers. After undergoing a thermal treatment and finishing process, a new supermetal with the trade name Gold-Wire was born. If we compare the Primary Wave One Gold file to its forerunner, the Primary Wave One M-Wire, we find that the former is at least 80% more flexible, the latter is 50% more resistant to cycle fatigue, and the latter is 23% more efficient [7]. All four folders have different names: red (25), green (35), white (45), and yellow (20) are the sizes from smallest (20/07) to largest (45/05). To prevent damage to the dentin, each file has a uniform taper from D1 to D3, followed by a taper with a progressively smaller percentage from D4 to D16.

So, this research was done to evaluate the differences between the dentinal defects caused by the Hyflex EDM, ProTaper Next, and Waveone Gold nickel-titanium files.

## Materials and methods

In order to conduct the study, 40 mandibular first premolars with single-rooted extracted teeth and straight canals, radiographically confirmed, were collected and maintained in filtered water. After using a diamond disc to remove the coronal parts, roots measuring around 16 mm in length were all that remained and were included in the study. Exclusion criteria included teeth with an open apex and roots that were examined for craze lines and cracks using a stereomicroscope at 12× magnification. These defects were all removed from the study's sample teeth and replaced with comparable teeth. Polyvinylsiloxane was used to coat the cemental surface of the roots to simulate the periodontal ligament space. Next, we inserted each root into its own acrylic block. The #10 K-File proved effective in opening up the blocked canal. Forty teeth were divided evenly among four groups of ten for the experiment, with each group receiving a different set of devices to use in preparation.

The untreated group (n = 10) served as the control in a study including canal preparation. Group 1 (n=10) employed Wave One Gold reciprocating files from Dentsply, Maillefer, Switzerland, with Xsmart Plus motors and a 6:1 reduction handpiece to clean and shape their canals. The canal was worked on using a size #10 file until it was fully loose after the working length was measured and a viscous chelator was applied. The Proglider was then installed to the recommended working length provided by the manufacturer. The interferences were removed by gently pressing down on the tip of the instrument with the main 25/08 Wave One Gold file and running the instrument 2, 3, and 4 mm inward in a brushing motion.

For the Protaper Next group (n=10), the procedure included inserting a #10 file into the canal and working on it in any spot until the channel was completely loose. This was accomplished in the presence of a thick chelator and with a working length that was roughly estimated. The Proglider was then installed to the recommended working length provided by the manufacturer. Instruments X1 (17/0.04) and X2 (25/0.06) were used in a continuous rotating action to enlarge the root canals until the WL was reached. This was done on both the buccolingual and mesiodistal extensions of each canal. We used an Xsmart Plus motor that generated 300 rpm and 2 Ncm of torque.

Using Xsmart Plus motors and a 6:1 reduction handpiece, the canals in the Hyflex EDM group (n=10) were prepared using the following sequence of Hyflex EDM files. The canal was worked on using a size #10 file until it was fully loose after the working length was measured and a viscous chelator was applied. After using a Hyflex EDM orifice opener to widen the opening, we followed the manufacturer's recommendations and inserted a Hyflex EDM glidepath file to the desired operating length. The canal was shaped to its full working length using a single Hyflex EDM file, rotated at 500 RPM with a torque of 2.5 Ncm, and moved gently in and out.

The photographs were taken with a digital camera mounted to the stereomicroscope at 12× magnification as the operators, who were blind to the technique, viewed the portions. Dentinal flaws (microcracks) were inspected in each specimen. A root dentin that has neither craze lines nor microcracks on either the internal or external surface of the root canal wall is said to have "NO DEFECT." If there were any fractures, lines, or microcracks in the root dentin, that was considered a "DEFECT."

The proportion of obviously faulty roots was compared between our control and experimental groups using a chi-square test conducted in SPSS/PC version 21 (SPSS, Inc., Chicago, IL).

## Results

Dentinal defects (microcracks) were evaluated in every sample. Each group had 30 different parts analyzed. In the coronal third region, the three experimental groups did not differ significantly from one another, as shown by the findings of the current investigation (Table 1).

**Table 1:** Coronal third region intergroup analysis

Coronal third	Control group (N %)	Protaper Next (N %)	Waveone Gold (N %)	Hyflex EDM (N %)	Total (N %)	P-value
No defect	10 (100%)	7 (70%)	7 (70%)	9 (90%)	33 (82.5%)	0.312
Defect	0 (0%)	3 (30%)	3 (30%)	1 (10%)	7 (17.5%)	

The middle-third comparison among the groups showed statistical significance (Table 2).

**Table 2:** Middle-third region intergroup analysis

Middle third	Control group (N %)	Protaper Next (N %)	Waveone Gold (N %)	Hyflex EDM (N %)	Total (N %)	P-value
No defect	10 (100%)	6 (60%)	7 (70%)	8 (80%)	31 (77.5%)	0.016
Defect	0 (0%)	4 (40%)	3 (30%)	2 (20%)	9 (22.5%)	

The apical third comparison among the groups showed no statistical significance (Table 3).

**Table 3:** Apical third region intergroup analysis

Apical third	Control group (N %)	Protaper Next (N %)	Waveone Gold (N %)	Hyflex EDM (N %)	Total (N %)	P-value
No defect	10 (100%)	7 (70%)	7 (70%)	8 (80%)	32 (80%)	0.076
Defect	0 (0%)	3 (30%)	3 (30%)	2 (20%)	8 (20%)	

Differences between the Protaper Next and Hyflex EDM groups were found to be statistically significant. Nonetheless, the Hyflex EDM cluster performed similarly to Waveone Gold.

## Discussion

Recently, there has been an explosion of innovative root canal preparation tools and methods thanks to the development of rotary nickel-titanium devices. Dentinal flaws, which may lead to vertical root fractures, are a serious risk with rotary nickel instrumentation (VRF) [7]. The instruments tend to come apart when made of nickel-titanium, which is another issue. Most cases of instrument separation may be traced back to cyclic fatigue or torsional fatigue [8]. Making Ni-Ti files with higher mechanical qualities via varied cross-sectional designs, surface treatment, and manufacturing procedures has been an ongoing endeavor by manufacturers to address the issue of instrument isolation and boost the versatility of Ni-Ti rotary instruments [9].

While using NiTi rotary instruments, the root canal walls are rotated at a high speed. The dentin of the root may develop microscopic fissures or craze lines as a consequence. The degree of this defect's production may depend on factors such as the design of the tip, the cross-section geometry, the kind of taper (constant vs. progressive), the pitch (constant vs. changing), and the form of the flute [8]. Dentine may separate from its attachments if forces generated inside the root canal are transmitted all the way to the root's surface [9]. Tensile tension in the canal wall causes fracture when it is greater than the dentine's ultimate tensile strength [10]. Hyflex EDM had a much lower mean value for the number of cracks in the apical third area than either Protaper Next or Waveone Gold.

Based on these findings, the middle part of the field is where Hyflex EDM and Protaper Next diverge significantly from one another. Comparing Hyflex EDM to Protaper Next, it had fewer cracks. There was no statistically significant difference between Hyflex EDM and Waveone Gold in terms of the number of cracks; however, Hyflex EDM did have fewer cracks overall. Flexible nickel-titanium instruments were shown to be responsible for the low number of fractures seen with Hyflex EDM in the apical third area of this investigation. Hyflex EDM has a greater degree of adaptability than the other two file systems [11], despite the fact that all three are heat-treated. This is likely due to the combined effects of the regulated memory wire and electric discharge machining.

In this study, the Hyflex EDM experienced a much lower rate of cracking in the investigation's middle third compared to the Protaper Next. All three files had a varying taper, although Protaper Next had more cracks than Hyflex EDM. One possible explanation is that the Protaper Next instruments are more finely tapered than their Hyflex EDM counterparts. This agrees with the findings of Adorno et al., who discovered that smaller tools were used to induce fractures more often than bigger ones [12]. While the difference between Hyflex EDM (continuous rotary) and Waveone Gold (reciprocating) in the number of cracks in the middle and apical third areas was not statistically significant, it was seen in the current investigation. For single-file instruments, the alloy from which they are made may have been more crucial than the speed of the instruments themselves in determining their destructive potential [13]. According to Yoldas et al. [14], cross-sectional file geometry may play a role in the development of dentinal cracks.

Although both Protaper Next and Waveone Gold have their benefits, the current investigation found that Hyflex EDM had the fewest cracks. Evidence like this suggests that factors other than instrument cross-sectional shape are more important in limiting the development of dentinal defects, such as the kind of alloy used, the degree of flexibility produced via heat treatment, and the quality of the manufacturing process. Proglider was utilized to generate glidepaths for both the Waveone Gold and Protaper Next groups in this investigation. In the Hyflex EDM treatment group, the glidepath was employed. In the central part of the sample, the fracture frequency was lowest for the Hyflex EDM glidepath files. Glidepath files, like their Hyflex counterparts, are made utilizing an electrical discharge machining (EDM) method using controlled memory wire technology [15,16]. The roots of the maxillary premolars, mandibular molars, and mandibular incisors are the most prone to fracture because their mesiodistal diameters are smaller than their buccolingual dimensions [17,18].

In the current study, limitations are that straight-canaled mandibular first premolars were selected because Hyflex EDM showed the fewest cracks in the middle third region. Hence, further research is required to assess the dentinal abnormalities brought on by Hyflex EDM in the most brittle roots, including those with a small mesiodistal diameter and a curved canal.

## Conclusions

The prognosis in endodontic therapy is positively correlated with optimal cleaning and shaping principles. Root canal shaping tools should make the most contact possible with the root canal walls for effective disinfection, and the remaining root structure should be sturdy and strong. Despite these advancements in technology, vertical root fracture and crack development continue to pose serious challenges for Ni-Ti instrument-based root canal shaping operations. Based on the data at hand, it seems that Hyflex EDM files fared better than Protaper Next and Waveone Gold when processed using the EDM technique. Careful consideration of a proper system is crucial to the long-term success of endodontists.
